# Health-Oriented Leadership and Mental Health From Supervisor and Employee Perspectives: A Multilevel and Multisource Approach

**DOI:** 10.3389/fpsyg.2020.614803

**Published:** 2021-01-18

**Authors:** Ruben Vonderlin, Burkhard Schmidt, Gerhard Müller, Miriam Biermann, Nikolaus Kleindienst, Martin Bohus, Lisa Lyssenko

**Affiliations:** ^1^Institute for Psychiatric and Psychosomatic Psychotherapy, Central Institute of Mental Health, University of Heidelberg, Mannheim, Germany; ^2^University of Applied Sciences Fresenius Heidelberg, Heidelberg, Germany; ^3^Department of Health Promotion/Occupational Health Management, AOK Baden-Wuerttemberg, Stuttgart, Germany; ^4^McLean Hospital, Harvard Medical School, Boston, MA, United States; ^5^Department of Public Health and Health Education, University of Education, Freiburg, Germany

**Keywords:** mutlilevel, multisource, health-oriented leadership, mental health, supervisor, employee

## Abstract

The link between leadership and mental health at the workplace is well established by prior research. However, most of the studies have addressed this relationship from a single-source perspective. The aim of this study was to examine how supervisor and employee ratings of health-oriented leadership correspond to each other and which sources are predictive for employee mental health. We assessed data within 99 teams (headed by 99 supervisors) containing 713 employees in 11 different companies in Southern Germany. Supervisors and their staff completed questionnaires on the supervisors’ health-oriented staff-care dimensions awareness, value of health and health behavior (Health-Oriented Leadership Scale, HoL) and current mental distress (Hospital Anxiety and Depression Scale, HADS). Hierarchical linear models revealed that supervisors’ self-ratings were significantly related to their employees’ ratings (at the team level) only on the health behavior dimension, but not on the health awareness and value of health dimensions. Also, supervisors rated themselves significantly higher on HoL compared to their employees. Employee ratings of HoL significantly predicted their own level of mental distress (direct within-level effect), whereas supervisor ratings of HoL did not predict employees’ mental distress at the team level (direct cross-level effect). Supervisors’ self-ratings of HoL did not influence the relationship between employee ratings of HoL and their mental distress on an individual level (cross-level interaction). These results highlight the complex relationship between multisource assessments of HoL and employee mental health, emphasizing the importance of subjective perception for mental health. Future studies should investigate under which conditions supervisor and employee ratings correspond to each other and are predictive for mental health at the workplace.

## Introduction

The potentially negative impact of job demands on employees’ (mental) health has been well established in previous research (Demerouti et al., 2001). Work demands can take many forms and are seen as physical, social and organizational aspects of the work that require sustained physical and/or psychological effort and are therefore associated with certain physiological and/or psychological costs (Demerouti et al., 2001). Well-studied examples are workload and work pressure, role conflicts or supervisor abuse. However, not only work demands can cause job strain, but employees who experience job strain also perceive more job demands over time (Bakker and Demerouti, 2017). Therefore, especially trough prolonged job demands, employees might enter a loss spiral of job demands and exhaustion and develop serious mental health impairments over time (e.g., Hakanen et al., 2008; Magnusson Hanson et al., 2016; Guthier et al., 2020). These impairments lead both to high individual suffering of those affected and to a considerable impairment of work performance and productivity (van den Heuvel et al., 2010; McTernan et al., 2013; Montano et al., 2017). Thus, the development of healthy workplaces is of central importance, as it is assumed to improve the health of employees, increase the productivity for the company and contribute to the wellbeing of the community at large (World Health Organization, 2005). Creating healthy workplaces and recognizing mental distress at an early stage, is therefore a central task for organizations and their representatives.

Supervisors have a special role to play here, as they bear corporate responsibility for employees in their daily working lives and influence their health in various ways (e.g., Kuoppala et al., 2008; Kelloway and Barling, 2010; Skakon et al., 2010). Supervisors design aspects of the work environment and work processes (Nielsen et al., 2008; Tuckey et al., 2012), pose demands or provide resources (Breevaart et al., 2014; Fernet et al., 2015), act as role models for their employees (Yaffe and Kark, 2011; Kranabetter and Niessen, 2017; Dietz et al., 2020) and directly interact with their employees through their leadership behavior and leadership style (Van Dierendonck et al., 2004; Montano et al., 2017; Inceoglu et al., 2018). Previous studies have shown that positive leadership behaviors and styles, such as appreciation, supervisor support, and transformational leadership, are particularly beneficial to the employees’ (mental) health, while negative leadership behaviors, such as supervisor abuse, can lead to a lasting impairment of employees’ (mental) health (e.g., Gilbreath and Benson, 2004; Judge and Piccolo, 2004; Hershcovis et al., 2007; Kuoppala et al., 2008; Stocker et al., 2010; Schmidt et al., 2014, 2018; Liao et al., 2018). Organizational theories and frameworks, like person-environment fit (Van Vianen, 2001), leader-member exchange (Dulebohn et al., 2012), or the job-demand resources model (Demerouti et al., 2001), agree on the point that individual perception of leadership behavior is a driver of the association between leadership and (mental) health in organizations (Harms et al., 2017). Therefore, models of “healthy leadership” are of growing interest in occupational health science (Rudolph et al., 2020). Although the empirical evidence to differentiate “healthy leadership” from other leadership styles is controversial (e.g., from transformational leadership; Dunkl et al., 2015; Rudolph et al., 2020), health-oriented leadership (HoL) concepts can be conceptualized as the supervisor’s direct and explicit engagement for the employees’ health (Gurt et al., 2011).

In this study, we build on the HoL concept by Franke et al. (2014). In their concept, they consider both the supervisor’s self-care, which is seen as a precondition for HoL, and the supervisor’s staff-care, which takes the employees’ health into focus. Self-care and staff-care can be further differentiated into the dimensions of health awareness, the value of health, and health behavior. In several studies, the positive relationship of HoL dimensions with mental health has been demonstrated (Franke et al., 2014; Klug et al., 2019; Santa Maria et al., 2019; Turgut et al., 2020). However, most of these studies have examined this relationship from a single-source (mostly from employee perspectives) in cross-sectional study designs. As a result, naturally occurring dynamics and possible crossover effects of HoL from different perspectives have been neglected, limiting any causal interpretation (Harms et al., 2017; Köppe et al., 2018). This common method bias (Podsakoff et al., 2003) might overestimate the effects of the predictors on the criterion variable, whereas other constructs might be artificially inflated, deflated, or non-significant (Podsakoff et al., 2012).

Although the requirement for multisource approaches to assess the impact of leadership on employee outcomes is not new (Conway and Huffcutt, 1997), only a few studies examined the impact of HoL on mental health from supervisors and employee perspectives (Köppe et al., 2018; Kaluza et al., 2020). The results of these studies are discussed later. Therefore, it remains unclear how supervisor ratings of their HoL are related to employee ratings and if supervisor ratings are predictive for employee mental health. To address these research questions (RQ), the first aim of the study was to test the relationship between supervisor and employee HoL ratings, and the second aim was to test which of these rating-sources (supervisors and their employees) are predictive for employee mental health.

### RQ1 How Are Supervisor and Employee Ratings Related to Each Other?

Multisource agreements are important indicators for psychometric assessments and reduce source errors (Strauss, 2005). Although multisource assessments for performance ratings are widely used in practice, the empirical evidence in terms of an interrater agreement between different rating sources is limited (Van Hooft et al., 2006). Meta-analytic findings estimated uncorrected correlations between supervisors and their subordinates on performance ratings at a level of 0.22 (supervisor-subordinate; Heidemeier and Moser, 2009). Furthermore, it has been assumed that correlations of different sources should be larger for observable patterns compared to non-observable constructs, as observable patterns show less ambiguity in ratings (Dai et al., 2007; Heidemeier and Moser, 2009; McKee et al., 2018). To date, there is scarce evidence of how multisource assessments of HoL are related to each other, specifically spoken whether the self-rating of supervisors in HoL corresponds to how they are seen by their teams. In line with prior findings on performance measures, we hypothesize that there should be a self-other agreement of HoL dimensions between supervisors and their employees on a team level that is significantly larger for the behavior dimension as an observable construct, compared to the awareness and value dimensions as non-observable constructs.

*Hypothesis 1:* Multisource agreement: Supervisor ratings of HoL are positively related to the employees’ averaged HoL ratings in each working team for the corresponding dimensions ([H-1a] awareness, [H-1b] value, [H-1c] behavior), with the largest relationship between behavior ratings.

However, a positive relationship between supervisor and employee ratings does not inform about whether or not self- and other ratings differ from each other. Prior research has shown that people tend to overestimate their own abilities, their performance, their chance of success, or their level of control (Meikle et al., 2016). This overestimation (often described as an operationalization of overconfidence bias) has been discussed as an adaptive process to protect one’s own self-esteem (Kahneman and Tversky, 1979) and is generally linked to mental health and well-being (Taylor and Brown, 1994; Dunning et al., 2004). Furthermore, it is assumed that this overestimation should be considerably larger if the respective ability is socially desirable (Alicke, 1985). As a general tendency in human beings, the overestimation bias should also be present at the workplace (Meikle et al., 2016). Prior research has shown that people in organizations are highly interested in how they are seen by others and might, for this reason, use methods of impression management, helping them to socially succeed (Leary and Kowalski, 1990; Stevens and Kristof, 1995). Indeed, an overconfidence bias has been shown to be related to the selection and hiring of supervisors and how their leadership competencies are perceived by others (Ronay et al., 2019). However, an overconfidence bias in supervisors has also been linked to negative or detrimental effects (e.g., aggressive or risky decision making; Malmendier and Tate, 2008). In line with these assumptions, Lee and Carpenter (2018) showed in a recent meta-analysis that supervisors report higher levels of their relationship-oriented behaviors than observers do. Atwater et al. (2005) have shown that supervisors rated themselves higher on performance ratings than their employees. In line with this empirical evidence, we propose that supervisors’ own HoL ratings should significantly exceed employee HoL ratings.

*Hypothesis 2:* Overconfidence-discrepancy: Supervisor ratings of HoL significantly exceed employee ratings ([H-2a] awareness, [H-2b] value, [H-2c] behavior).

### RQ2 Which Source Is Predictive for Mental Health?

In addition to the well established relationship between single-source ratings of HoL and mental health on the employee level, the question has been raised whether supervisor self-ratings are also predictive for employee mental health (direct cross-level effect; Franke et al., 2014). The extant literature on multisource assessment in HoL is limited. To our knowledge, only one study has investigated a multisource cross-level effect of HoL on employee mental health, showing a significant relationship between supervisors’ health awareness and behavior with employees’ exhaustion (Kaluza et al., 2020). Furthermore, previous research has shown that the quality of leader-member relationships rated by supervisors is positively associated with feeling respected and supported by employees, but not with their level of perceived stress (Chen and Tjosvold, 2013). The relationship between supervisor-rated transformational leadership and employee job stress was small but statistically significant (Sosik and Godshalk, 2000). However, most of these constructs reflect proximal health outcomes at the workplace, like job stress, job-related exhaustion or organizational support. To date, it remains unclear whether supervisor behaviors also have beneficial effects on more distal outcomes, like general mental distress. Based on previous findings, we propose that both supervisor and employee ratings of HoL significantly predict employee mental health, with larger effects for single-source ratings (i.e., employee ratings).

*Hypothesis 3:* Direct-within-level effect: Employee ratings of HoL are negatively related to their individual anxiety and depression symptoms ([H-3a] awareness, [H-3b] value, [H-3c] behavior).

*Hypothesis 4:* Direct-cross-level effect: Supervisor ratings of HoL are negatively related to the employees’ averaged anxiety and depression symptoms at the team level ([H-4a] awareness, [H-4b] value, [H-4c] behavior), i.e., supervisors with high HoL staff-care self-ratings have healthier working teams.

### RQ3 Do Supervisor Ratings Influence the Relationship Between Employee Ratings and Mental Health?

It remains unclear whether HoL ratings from supervisors influence the relationship between employees’ HoL ratings and their mental health. Different psychological theories, like person-environment fit (Van Vianen, 2001), leader-member exchange (Dulebohn et al., 2012) or social learning theory (Bandura, 1986) agree on the point that employee and supervisors perceptions of leadership are highly intercorrelated and interactive in nature. In a literature review of healthy leadership Rudolph et al. (2020) theoretically suggested that leader individual differences might function as a moderator of the relationship between perceived HoL and health outcomes. Indeed several studies have demonstrated cross-level interactions in the leadership literature (also from a multisource perspective), which describe whether relationships between the lower-level variables change as a function of higher-order moderator variables (Aguinis et al., 2013). For example it was shown that the relationship between transformational leadership and mental distress on an individual employee level was moderated by how the supervisor assessed his or her own health awareness (Kranabetter and Niessen, 2017). In addition, it was shown that supervisors’ perceived organizational support moderated the relationship between leader-member exchange and job satisfaction of employees (Erdogan and Enders, 2007). Applied to our study, this perspective raises the question whether supervisors’ self-ratings of HoL could further enhance the positive effects of employee rated HoL on their mental health. Since the investigation of moderators on the relationship between leadership and health has been stated as an important direction for future research (Rudolph et al., 2020) and the use of multisource ratings was recommended to assess organizational aspects as possible moderator variables (Inceoglu et al., 2018), we tested whether supervisor ratings of HoL influence the relationship between employee ratings of HoL and their mental health. Therefore, in Hypothesis 5, we propose the following cross-level interaction hypothesis:

*Hypothesis 5:* Cross-level interaction: The relationship between employee HoL ratings and their individual mental health is stronger in teams with supervisors scoring high on the respective HoL-dimensions compared to teams with supervisors scoring low on the respective HoL-dimensions ([H-5a] awareness, [H-5b] value, [H-5c] behavior).

## Materials and Methods

### Participants and Procedure

The data were gathered from 11 companies from different branches in Southern Germany. The study was approved by the ethical review committee at the University of Heidelberg, 2017562NMA. Participation in the study was voluntary, and informed consent was obtained. A trustee assigned a study code to all participants, irrespective of their organization and the scientific staff, which contained information about the employees’ affiliation to their supervisors as well as to the company. Employees completed a questionnaire that assessed supervisors’ HoL staff-care and their own mental distress. Supervisors completed a questionnaire that assessed their HoL staff-care as a self-rating instrument. All the participants who returned their questionnaire had the opportunity to enter a prize draw to win one of 40 vouchers worth €20 each for an online store. In total, 1,731 employees and 137 supervisors across the companies were contacted to participate in our study. A response rate of 46% for employees and 95% for supervisors yielded a sample of 803 employees and 130 supervisors who completed questionnaires. In order to obtain reliable results for the comparison between employees’ and supervisors’ perspectives, we included only those participants in our analyses for whom data was provided at both levels (data from employees and supervisors available). Therefore, we built up a data set matching employees to their supervisors. After the matching procedure, 734 employees and 111 supervisors remained in the sample. Due to the multilevel structure that comprises individuals in teams, we did not include supervisors matched with only one employee in the analyses and reduced the sample to teams with at least two employees. This left us with a final sample of 713 employees and 99 supervisors. The employees averaged 41.40 years of age (*SD* = 12.67); the supervisors averaged 47.68 years of age (*SD* = 9.13). Most (71.0%) of employees and 48.5% of supervisors were female. The average team size was 13.70 employees (SD = 12.60; range = 1–83). The average group number of respondents from participating workgroups was 7.20 employees (SD = 5.34; range = 2–29 employees). The percentage of respondents holding a high-school graduation degree was 43% for employees and 52% for the supervisor. From the 11 companies, three nursing homes, three hospitals, two manufacturers of parts and accessories for motor vehicles, one recreation and holiday home, one waste management company, and one research and development company, participated.

### Assessments

#### Health-Oriented Leadership

Health-oriented leadership was assessed with Franke et al. (2014) HoL instrument. The HoL instrument consists of different scales measuring supervisors’ staff-care, as well as supervisors’ and employees’ self-care. All the scales include the three dimensions *health awareness* (eight items), *value of health* (three items), and *health behavior* (14 items) on a five-point Likert-Scale. Parallel versions exist for all scales, which can be used both for self-assessment by supervisors and for external assessment by employees. Thus, the questionnaire can be used to conduct multisource assessments. The good psychometric properties of the scales have been demonstrated, showing good internal consistencies (α = 0.84 to 0.88) as well as high construct and criterion validity (Franke et al., 2014). In this study, we assessed the supervisors’ staff-care from supervisor and employee perspectives. Thus supervisors’ had to rate their own health awareness (e.g., “I immediately notice when something is wrong with my employees’ health”), value of health (e.g., “It is important for me to reduce health risks at my employees’ workplaces”), and health behavior (e.g., “I invite my employees to inform me about health risks at their workplaces”) toward their employees. In addition employees were asked to rate their supervisors’ health awareness (e.g., “My supervisor immediately notices when something is wrong with my health”), value of health (e.g., “It is important for my supervisor to reduce health risks at my workplace”), and health behavior (e.g., “My supervisor invites me to inform him/her about health risks at my workplace”). Cronbach’s α in our sample ranged from 0.88 to 0.92 for staff-care assessed by employees and 0.77 to 0.85 for staff-care assessed by supervisors, indicating good internal consistencies for the construct measured.

#### Mental Distress

Mental distress was measured with the Hospital Anxiety and Depression Scale (HADS; Herrmann-Lingen et al., 2011). The HADS consists of two subscales measuring symptoms of *depression* (7 items) and *anxiety* (7 items) on a four-point Likert-Scale. The psychometric properties of the HADS show good internal consistency (Cronbach’s α = 0.80) as well as a good construct and criterion validity. Cronbach’s α in our sample was 0.90 for employees and 0.86 for supervisors indicating good internal consistencies for the construct measured. Due to a high level of acceptance in non-clinical samples, the HADS is internationally used as a screening instrument for mental disorders (Bjelland et al., 2002).

### Statistical Analyses

To account for the hierarchical structure of the data (employees [level 1] nested within supervisors [level 2]), we analyzed H-1 and H-3 to H-5 using mixed-effects models. According to theoretical assumptions, HoL rated by the employees represents the individual perception of each employee and was included as a predictor on the individual (team member) level. In contrast, supervisor ratings of their own HoL were included as a team level predictor because it influences the team as a whole. Thus, we used no aggregation methods to reflect the higher-order constructs of the predictor variables. Regarding the dependent variables we analyzed HoL assessed by employees on the team level to analyze whether supervisor self-ratings correspond with their team ratings (H-1). Mental distress was analyzed both, on the individual level (H-3) as well as on the team level (H-4). As previous research has shown that leadership styles might vary according to age and gender of supervisors (e.g., Eagly and Johnson, 1990; Burke and Collins, 2001), we included age and gender of employees as control variables at level 1 as well as age and gender of supervisors as control variables at level 2. All analyses were performed in R with the nlme package (Bliese, 2016; Pinheiro et al., 2020) according to the guidelines of Raudenbush and Bryk (2002).

To analyze H-1 (agreement of supervisor and employee ratings), we conducted a two-step model-building process: The first step contained the null model with employee HoL ratings as dependent variables to analyze intraclass correlations [ICC(1)s, i.e., the percentage of variance that can be explained by group membership] of HoL ratings on the team level. The second step contained a random intercept and fixed slope model including the control variables as well as supervisor HoL ratings as level 2 predictors to predict the employees’ averaged HoL ratings in the working teams. To test H-2 (overconfidence-discrepancy), we compared supervisor and employee ratings of HoL using independent sample *t*-tests. To analyze H-3 to H-5, we carried out a four-step model building process with employees’ mental health as dependent variable and employee (level 1) and supervisor (level 2) HoL ratings (awareness, value and behavior) as predictors. The first step contained the null model; the second step contained a random intercept and fixed slope model including the control variables as well as analyzing the effects of employee HoL ratings on their individual mental health (direct within level effect; H-3) and the effects of supervisor HoL ratings on the averaged mental health in the respective working team (direct cross-level effect; H-4); the third step contained a random intercept and random slope model to assess the variance of slopes across teams; finally, in the fourth step we conducted the cross-level interaction model to analyze whether supervisors HoL ratings moderate the relationship between employees’ HoL ratings and their individual mental health across teams (H-5). In terms of centering, we used grand-mean centering for level 2 (supervisor level) variables. On level 1, we assumed that the absolute HoL ratings of employees are more important for their mental health as their relative position within their teams. In line with Hox (2002) we, therefore, used grand-mean centering for level 1 variables.

Since we collected data within 11 different companies, the hierarchical data structure might imply possible company level effects. To address these, ICCs of outcomes on the company level were analyzed accordingly. Results showed ICCs < 0.05 for all employee outcomes at the company level (0.03 for awareness, 0.04 for value and 0.04 for behavior, 0.00 for depression, and 0.00 for anxiety). The ICCs of supervisors’ HoL at the organizational level were comparable low (0.00 to 0.06). Given the low ICCs and to furthermore reduce the complexity of the statistical models to enhance parameter estimation, we decided not to include the company as an additional level in the models.

The high intercorrelations between the three HoL subscales awareness, value, and behavior (0.74 to 0.78) and between the HADS scores of depression and anxiety (0.93) indicate a considerable overlap of the respective subscales and a risk of stochastic multicollinearity (see Table 1). Conceptually, it can be assumed that the HoL subscales are strongly correlated, but still represent different dimensions of the construct. Therefore, we first analyzed the condition index (CI) to assess whether a robust estimation of model parameters is feasible (according to common interpretation guidelines, a CI score <30 indicates low collinerarity and a robust estimation of the model parameters; Belsley et al., 1980). In addition, we analyzed the tolerance values of the HoL subscales. Tolerance values are defined as 1-*R*^2^ and represent the amount of variability in one independent variable that is not explained by the other independent variables (according to common interpretation guidelines a tolerance value <0.40 suggests a cause for concern, whereas a tolerance value <0.20 suggests serious stochastic multicollinearity in a model; Allison, 1999; Weisburd and Britt, 2014). To verify the three-factor solution of the HoL scales and the two-factor solution of the HADS scales, we performed confirmatory factor analyses (CFA). Analyses revealed a CI of 3.62 for HoL subscales. Tolerance values ranged from 0.33 for awareness and value to 0.37 for behavior. CFA analyses of the HoL scales showed that the three-factor model (*χ^2^* = 1212, df = 167, RMSEA = 0.09, CFI = 0.90, SRMR = 0.06), fitted the data significantly better than the one-factor solution [Δχ^2^(3) = 1655, *p* < 0.001]. Thus, although the different subscales of HoL were highly correlated, we decided to use the subscales instead of building a global mean score. However, given the high intercorrelations, we decided to model them separately from each other, in order to avoid masking effects by having multiple interaction terms in the statistical models and to improve the interpretability of the results. CFA analyses of the HADS scales showed that the proposed two-factor model (*χ^2^* = 510, df = 76, RMSEA = 0.09, CFI = 0.90, SRMR = 0.06), fitted the data significantly better than the one-factor solution [Δχ^2^(1) = 75, *p* < 0.001]. Therefore we decided to analyze the depression and anxiety scales separately from each other, instead of building a global mental distress score.

**TABLE 1:** Summary of employees’ (level 1) and supervisors’ (level 2) multi-source intercorrelations, means, and standard deviations, and ICCs on team and organizational level.

Measure	Mean	SD	ICC	1	2	3	4	5	6	7	8	9	10	11	12	13
**Level 1 (employee ratings)**																
1. Age	41.40	12.67	0.17	1	0.10*	−0.16**	−0.14**	−0.14**	0.13*	0.04	0.15**	0.10**	–0.05	0.04	0.07	0.12**
2. Gender^a^	71.00	–	0.51		1	0.02	–0.04	–0.01	−0.09*	0.01	0.02	0.43**	0.14**	0.08*	0.17**	0.24**
3. HoL awareness	3.02	1.00	0.22			1	0.78**	0.75**	−0.42**	−0.32**	–0.06	0.10**	0.08*	0.04	0.07	−0.10**
4. HoL value	3.24	1.13	0.27				1	0.75**	−0.38**	−0.29**	–0.05	–0.01	0.08*	0.06	0.10**	−0.11**
5. HoL behavior	2.51	1.05	0.23					1	−0.33**	−0.24**	–0.01	0.05	0.12**	0.04	0.18**	–0.04
6. Depression	4.65	3.72	0.08						1	0.93**	0.04	−0.08*	–0.06	0.04	–0.03	0.02
7. Anxiety	6.80	3.85	0.04							1	0.04	–0.03	0.00	0.02	0.00	0.01
**Level 2 (supervisor ratings)**																
8. Age	47.68	9.13	0.00								1	0.03	–0.06	–0.02	0.32**	0.01
9. Gender^a^	48.50	–	0.17									1	0.27**	0.05	0.30**	–0.02
10. HoL awareness	3.92	0.54	0.06										1	0.34**	0.44**	0.08*
11. HoL value	4.54	0.60	0.02											1	0.40**	0.07*
12. HoL behavior	3.52	0.75	0.00												1	0.22**
13. Team size^b^	2.26	0.65	–													1

Level 1 variables represent employee ratings (*n* = 713), Level 2 variables represent supervisor ratings (*n* = 99). Correlations are based on the individual employee level. ^*a*^% female, gender was coded with 0 = male, 1 = female. ^*b*^Team size was assessed categorical (1 < 5 employees, 2 = 5–20 employees, 3 > 20 employees). ICCs on level 1 represent intraclasscorrelations on team level. ICCs on level 2 represent intraclasscorrelations on organizational level. *p < 0.05, **p < 0.01, ***p < 0.001.

## Results

The descriptive statistics, ICCs and intercorrelations of all variables on the individual employee level are presented in Table 1. We detected significant positive intercorrelations of the HoL dimensions on the employee and supervisor level. Employee HoL ratings showed significant negative intercorrelations with their mental distress. Interestingly, a younger age of the employees was associated with higher HoL ratings from their own perspective. In addition, supervisor female gender was associated with higher ratings of employees’ HoL awareness and higher ratings of supervisors’ HoL awareness and behavior. ICCs of 0.22 to 0.27 for employee HoL ratings indicate that 22 to 27% of the variability is accounted for by team membership. For mental distress, ICCs were considerably smaller and ranged from 0.04 to 0.08, indicating that only 4 to 8% of the variability in mental distress is accounted for by team membership. Overall, these results show evidence for a nested data structure that requires multilevel modeling.

### Testing of Hypotheses

To test hypothesis 1, we conducted a two-step model building process. In step 1, we analyzed the ICCs of the HoL dimensions awareness, value and behavior, which are depicted in Table 1. As shown in Table 2, the across team variances of the HoL dimensions ranged from 0.222 to 0.348 and the within team variances ranged from 0.782 to 0.945. In step 2, we included age and gender as control variables as well as supervisor ratings of the corresponding dimensions to predict the averaged employee ratings in the working teams to assess the multisource agreement. We found no significant multisource agreement for awareness and value (awareness, *Υ_01_* = 0.088, *p* = 0.440; value, *Υ_01_* = 0.158, *p* = 0.234). However, the multisource agreement for behavior yielded significance (*Υ_01_* = 0.234, *p* = 0.011). Thus, only Hypothesis 2c was supported, and Hypotheses 2a and 2b were not.

**TABLE 2:** Results from multilevel modeling analyses for supervisor ratings predicting employee ratings on the HoL dimensions (multi-source agreement).

	HoL awareness^1^	HoL value^2^	HoL behavior^3^
**Level 1 (employee ratings)**			
Intercept	3.037 (0.061)***	3.267 (0.073)***	2.498 (0.064)***
Employees’ age	−0.013 (0.003)***	−0.011 (0.003)***	−0.011 (0.003)***
Employees’ gender	0.017 (0.094)	0.020 (0.106)	−0.015 (0.098)
**Level 2 (supervisor ratings)**			
Supervisors’ age	−0.005 (0.007)	−0.008 (0.008)	−0.007 (0.007)
Supervisors’ gender	0.182 (0.132)	−0.045 (0.155)	0.004 (0.134)
Health awareness^1^/value^2^/behavior^3^	0.088 (0.114)	0.158 (0.132)	0.234 (0.090)*
**Variance components**			
Within-team (L1) variance	0.782	0.945	0.837
Intercept (L2) variance	0.222	0.348	0.246

HoL, health-oriented leadership; L1, level: *n* = 713 1; L2, level 2: *n* = 99. To assess multi-source agreement, we used ^1^awareness (L2) to predict awareness (L1), ^2^value (L2) to predict value (L1), and ^3^behavior (L2) to predict behavior (L1). Values in parentheses are standard errors. *p < 0.05, ***p < 0.001.

**FIGURE 1:**
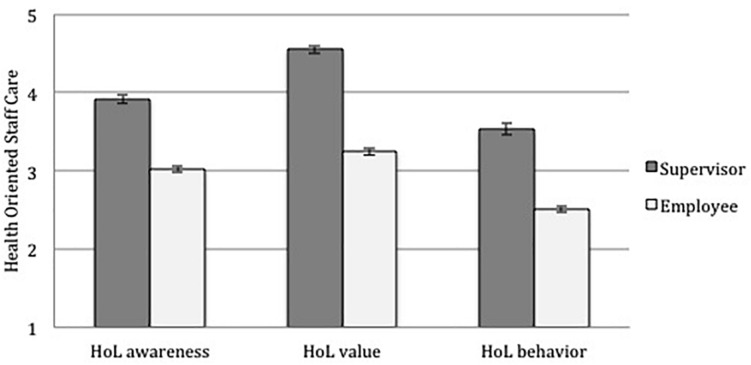
HoL ratings from supervisors and their employees; given are the means; error bars indicate the standard error of the mean.

To test hypothesis 2, we compared employee HoL ratings with supervisor self-HoL ratings. The mean scores on employee level yielded *M* = 3.02 (*SD* = 1.00) for awareness, *M* = 3.24 (*SD* = 1.13) for value, and *M* = 2.51 (*SD* = 1.05) for behavior. Supervisors’ ratings significantly exceeded employee rating on all dimensions with *M* = 3.92 (*SD* = 0.54) for awareness [*t*(810) = 8.78, *p* < 0.001), *M* = 4.54 (*SD* = 0.60] for value [*t*(810) = 11.23, *p* < 0.001] and *M* = 3.52 (*SD* = 0.75) for behavior [*t*(810) = 9.25, *p* < 0.001]. Descriptive statistics are presented in Figure 1. Hence hypothesis 2 was confirmed showing that supervisor ratings of HoL significantly exceed employee ratings.

To analyze H-3 to H-5, we carried out a four-step model building process. In step 1, we calculated the ICCs of mental distress. As shown in Table 3, the across team variance of depression was 1.120 and the within team variance was 12.682, whereas the across team variance of anxiety was 0.610, and the within team variance was 14.220. In step 2, we included age and gender as control variables and analyzed the direct within and cross level effects. We tested the direct within level effect (H-3) by including employee HoL ratings as level 1 predictors. There was a significant relationship of employees’ HoL ratings of health awareness with their individual depression and anxiety symptoms (depression: *Υ_10_* = −1.456, *p* < 0.001; anxiety *Υ_10_* = −1.248, *p* < 0.001). In addition, we found a significant relationship of employees’ HoL ratings of health value with their individual depression and anxiety symptoms (depression: *Υ_10_* = −1.218, *p* < 0.001; anxiety: *Υ_10_* = −0.995, *p* < 0.001), as well as a significant relationship of employees’ HoL ratings of health behavior with their individual depression and anxiety symptoms (depression: *Υ_10_* = −1.114, *p* < 0.001; anxiety: *Υ_10_* = −0.894, *p* < 0.001). Thus, hypotheses 3a–3c were supported.

**TABLE 3:** Results from multilevel modeling analyses for supervisor and employee ratings of supervisors’ health awareness, value and behavior predicting depression and anxiety symptoms.

	Depression	Anxiety
**Model 1 Health awareness**		
**Level 1 (employee ratings)**		
Intercept	4.704 (0.180)***	6.183 (0.169)***
Employees’ age	0.023 (0.011)*	−0.004 (0.011)
Employees’ gender	−0.657 (0.321)*	0.148 (0.345)
Health awareness	−1.456 (0.132)***	−1.248 (0.143)***
**Level 2 (supervisor ratings)**		
Supervisors’ age	0.008 (0.016)	0.012 (0.017)
Supervisors’ gender	−0.093 (0.334)	−0.074 (0.355)
Health awareness	0.006 (0.281)	0.223 (0.298)
**Cross-level interaction**		
Health awareness × Health awareness	0.173 (0.238)	−0.105 (0.255)
**Variance components**		
Within-team (L1) variance	12.682	14.220
Intercept (L2) variance	1.120	0.610
Slope (L2) variance	0.210	0.274
Intercept-slope (L2) covariance	−0.285	−0.046
**Model 2 Health value**		
**Level 1 (employee ratings)**		
Intercept	4.704 (0.180)***	6.183 (0.169)***
Employees’ age	0.027 (.011)	−0.001 (.011)
Employees’ gender	−0.725 (0.325)	0.107 (0.350)
Health value	−1.218 (0.118)***	−0.995 (0.127)***
**Level 2 (supervisor ratings)**		
Supervisors’ age	0.008 (0.016)	0.020 (0.017)
Supervisors’ gender	−0.421 (0.332)	−0.294 (0.349)
Health value	0.502 (0.300)	0.328 (0.318)
**Cross-level interaction**		
Health value × Health value	0.343 (0.281)	0.497 (0.303)
**Variance components**		
Within-team (L1) variance	12.682	14.220
Intercept (L2) variance	1.120	0.610
Slope (L2) variance	0.248	0.269
Intercept-slope (L2) covariance	−0.288	−0.111
**Model 3 Health behavior**		
**Level 1 (employee ratings)**		
Intercept	4.704 (0.180)***	6.183 (0.169)***
Employees’ age	0.029 (0.011)*	0.001 (0.012)
Employees’ gender	−0.707 (0.333)	0.111 (0.355)
Health behavior	−1.114 (0.132)***	−0.894 (0.141)***
**Level 2 (supervisor ratings)**		
Supervisors’ age	0.009 (0.018)	0.019 (0.018)
Supervisors’ gender	−0.360 (0.352)	−0.268 (0.368)
Health behavior	0.256 (0.242)	0.281 (0.254)
**Cross-level interaction**		
Health behavior × Health behavior	−0.154 (0.191)	0.197 (0.333)
**Variance components**		
Within-team (L1) variance	12.682	14.220
Intercept (L2) variance	1.120	0.610
Slope (L2) variance	0.319	0.252
Intercept-slope (L2) covariance	−0.239	−0.069

HoL, health oriented leadership; L1, level 1: *n* = 713 employees; L2, level 2: *n* = 99 supervisors (teams). Values in parentheses are standard errors. *p < 0.05, ***p < 0.001.

We tested the direct cross level effect (H-4) by including supervisor HoL ratings as level 2 predictors. Results showed no significant relationship of supervisors’ ratings of health awareness with employees’ averaged mental distress in working teams (depression: *Υ_01_* = 0.006, *p* = 0.982; anxiety: *Υ_01_* = 0.223, *p* = 0.456), no significant relationship of supervisors’ ratings of health value with employees’ averaged mental distress in working teams (depression: *Υ_01_* = 0.502, *p* = 0.098; anxiety: *Υ_01_* = 0.328, *p* = 0.304), as well as no significant relationship of supervisors’ ratings of health behavior with employees’ averaged mental distress in working teams (depression: *Υ_01_* = 0.256, *p* = 0.294; anxiety: *Υ_01_* = 0.281, *p* = 0.272). Thus, hypotheses 4a–4c were not supported. In step 3, we examined the slope variation of employee HoL ratings in the prediction of depression and anxiety, as the slope variance is a precondition for examining cross-level moderators (H-5). The variance in slopes of employees’ health awareness across groups when supervisors’ health awareness was included in the model is 0.210 for depression and 0.274 for anxiety. The variance in slopes of employees’ health value across groups when supervisors’ health value was included in the model is 0.248 for depression and 0.269 for anxiety. The variance in slopes of employees’ health behavior across groups when supervisors’ health behavior was included in the model is 0.319 for depression and 0.252 for anxiety. Hence, results demonstrate only small group differences for the relationship of employees’ HoL ratings with employee depression and anxiety. Thus, an interaction effect seems unlikely. In Step 4, we aimed to test whether health awareness, value, and behavior of supervisors moderates the relationship of employee ratings of health awareness, value, and behavior with their depression and anxiety (Hypotheses 5a–5c). Results do not support Hypotheses 5a–5c, suggesting no significant cross-level interaction effect of supervisors’ HoL ratings. The negative relationship between employee health awareness with depression and anxiety is not affected by supervisors’ rating of health awareness (depression: *Υ_11_* = 0.173, *p* = 0.467; anxiety: *Υ_11_* = −0.105, *p* = 0.680). The negative relationship between employee health value and depression and anxiety is not affected by supervisors’ rating of health value (depression: *Υ_11_* = 0.343, *p* = 0.222; anxiety: *Υ_11_* = 0.497, *p* = 0.101). Finally, the negative relationship between employee health behavior and depression and anxiety is not affected by supervisors’ rating of health behavior (depression: *Υ_11_* = −0.154, *p* = 0.419; anxiety: *Υ_11_* = 0.197, *p* = 0.333). Thus, hypotheses 5a–5c were not supported.

## Discussion

The principal aim of the current study was to examine whether and to what extend HoL ratings from supervisors and employees correspond to each other and how they are linked to employees’ mental health. Based on the concept of HoL (Franke et al., 2014), we assessed health awareness, the value of health, and the health behavior of supervisors from the supervisor and employee perspectives. We found that supervisor and employee ratings of HoL only correspond on the “behavior dimension,” but not on the dimension “value of health” and “health awareness”. The employee ratings of HoL were predictive for their mental health, but supervisor ratings were not. Also, no interaction effect was found, indicating that supervisor ratings of HoL did not influence the relationship between employee ratings of HoL and their mental health on an individual level.

The analysis of the ICCs at the employee level showed higher scores on the HoL dimensions than on the mental health of employees. Overall, 22–27% of the variance in HoL was explained by team membership, compared with only 7–8% for mental health. The ICCs for the different dimensions of HoL were comparable. These results suggest that the evaluation of leadership is more dependent on team membership than mental health. Previous studies have shown similar ICCs for health in teams of 0–10% (Kranabetter and Niessen, 2017) and leadership constructs of 20–23% (Zwingmann et al., 2014).

Intercorrelations on the individual employee level showed that younger age of the employees was associated with higher HoL ratings from their own perspective. In addition, the female gender of supervisors was associated with higher ratings of employees’ HoL awareness and higher ratings of supervisors’ HoL awareness and behavior. This finding suggests a gender-specific effect on HoL that is congruent with previous findings on gender differences in leadership style. For example, prior research has shown that women have a more interactive and participative and less autocratic and directive leadership style than men (Eagly and Johnson, 1990; Burke and Collins, 2001). However, these correlative results are only a first indicator of a gender-specific effect on HoL. This effect should be further investigated in future research to enable reliable conclusions to be drawn.

With regard to the self-other agreement, the multilevel analyses did not reveal significant relationships between awareness and value of health between supervisor and employee ratings (thus, hypotheses 1a and 1b were not confirmed). Thus the supervisors’ ratings of their own HoL awareness and value were not related to the average HoL ratings from their working teams. However, on the behavioral dimension, a significant relationship was obtained between the two sources (thus, hypothesis 1c was confirmed). Accordingly, supervisors who rated themselves highly in HoL behavior were also rated highly by their work teams. This could be due to the fact that certain supervisor behaviors can be observed from third parties, while the dimensions of health awareness and value of health are not directly observable – therefore showing less ambiguity in behavior ratings (Dai et al., 2007). This is in line with current guidelines to assess psychological constructs from an inter-rater perspective, in which behaviorally anchored scales are widely used (Ohland et al., 2012).

Furthermore, the results of the study show that supervisors’ HoL ratings significantly exceed their employees’ ratings on all dimensions. Accordingly, supervisors rated themselves to be more aware of employee health, to have a higher value of health, and to show more health behaviors, in comparison to the assessments of their employees (thus, hypotheses 2a–2c were confirmed). These results are consistent with previous findings in the overconfidence-bias literature (Kahneman and Tversky, 1979) and impression management literature (Leary and Kowalski, 1990) showing higher ratings of supervisors on their abilities and performance measures compared to their followers (Atwater et al., 2005; Lee and Carpenter, 2018). However, it can be assumed that this discrepancy is not specific to the workplace, but rather represents a general psychological tendency that also occurs outside the workplace. As the absence of overconfidence bias has been linked to depressive symptoms (Korn et al., 2014), supervisors might profit from optimistically biased beliefs in various areas, such as mental health, job performance, or social interactions.

An examination of the relationship between HoL and mental health showed that only the subjective assessments of employees significantly predicted their mental health (thus, hypotheses 3a–3c were confirmed). However, the supervisors’ assessments of their HoL showed no significant effect on the averaged health of employees in the working teams (thus, hypotheses 4a–4c were not confirmed). On the one hand, this shows that the subjective perception (appraisal) of HoL plays a central role in stress processing, which is in line with Lazarus and Folkman (1984) stress appraisal model. On the other hand, this result raises the question of how and under which conditions supervisor ratings of HoL are related to the mental health of employees and underlines the complex nature of the link between leadership and mental health (Harms et al., 2017). However, the small ICC values (0.04 to 0.08) of employees’ mental distress represent an important limitation to the interpretability of these findings (LoPilato et al., 2014). Usually, a high within-group agreement on mental distress would be needed, to establish the effect of supervisor HoL ratings (as a higher-level construct) on the group average of mental health. However, the small ICCs in our sample could indicate a rather low that the within-group mean of mental health is a good representation of all individuals in a group. In that case, the interpretation of the statistical cross-level direct effect as the theoretical cross-level direct effect could be considered problematic. At which level of an ICC, a multilevel analysis should be considered, is however a controversial discussion (Aguinis et al., 2013). For example, even low ICCs of 0.10 or even 0.05 might suggest, that a level 2 variable explains heterogeneity of the dependent variable across teams (Peugh, 2010; Kahn, 2011). In conclusion, there are no consistent ICC size thresholds in multi-level analyses. In the light of this discussion, the multilevel results on mental health should be interpreted with caution.

Finally, our results show only small differences in slopes for the relationship of employees’ HoL ratings with their own depression and anxiety across the groups. Consequently, we found no significant interaction effect between the supervisor and employee HoL ratings on the mental health of employees (thus, hypotheses 5a–5c were not confirmed). This finding shows that the mental distress of employees was related to their own perception of HoL, but this relationship did not vary depending on the supervisors’ assessment.

Further research is needed to understand the complex and still unclear relationship between supervisor assessments, employee assessments, and the health effects of the two sources. Furthermore, it is important to understand when and under which conditions the assessments between supervisors and employees correspond to each other and under which conditions supervisor assessments affect the health of employees. Previous research has shown that self-other agreement might be dependent from contextual work characteristics (Ostroff et al., 2004), cultural aspects (Cullen et al., 2015) as well as personal characteristics of the supervisors, such as age, gender or personality (Bergner et al., 2016; McKee et al., 2018). Also, other leadership styles, such as authentic leadership, could moderate this relationship, which can be roughly characterized by a high degree of authenticity, self-confidence and self-regulation, transparency, and honesty of supervisors (Gardner et al., 2011). This complexity of the relationship between leadership behavior and health was demonstrated in a single-source study in which role ambiguity and climate of learning mediated the effect between leadership behavior and health, this mediation being further moderated by job autonomy (Berger et al., 2019). Future studies should also apply such mediator and moderator analyses in multisource studies. In addition to assessments of the leadership behavior and style by supervisors and employees, the attitudes of supervisors toward their employees [e.g., whether supervisors are able to take the perspectives of their employees; Gregory et al. (2011)] and the attitudes of employees toward their supervisors should also be assessed [e.g., trust in supervisors; Stedham and Skaar (2019)]. This could contribute to a better understanding of the complex relationship between leadership and health.

### Limitations and Recommendations for Future Research

Although our study contributes to the further understanding of the relationship between supervisor and employee rated HoL and employee mental health from a multisource perspective, it has some limitations that should be mentioned.

First, we used a cross-sectional study design, which clearly limits the degree to which we could make causal inferences regarding the relationships of HoL and mental health. The findings of the present study should be replicated by longitudinal or experimental designs to test the causal relationship of HoL and mental health.

Second, the small ICC values of employees’ mental distress represent an important limitation to the interpretability of the cross-level findings, because it raises the question whether the group mean of mental distress is a good representation of all individuals in the group.

Third, we built our research study on the HoL concept from Franke et al. (2014), as it is one prominent and broad framework that helps to conceptualize HoL. We decided to use the concept of HoL, because it reflects the direct engagement of supervisors in their employees’ health (Gurt et al., 2011) and it provides a psychometrically proofed questionnaire that can be used for multisource assessment purposes. In previous literature, however, the construct proliferation and the confusion of different HoL approaches have been criticized (Rudolph et al., 2020). Other approaches, e.g., broader concepts looking on different aspects of several behaviors like task-oriented, relationship-oriented, change-oriented, and passive/destructive behaviors (Wegge et al., 2014; Inceoglu et al., 2018) or other health-beneficial leadership styles (e.g., transformational leadership), can therefore add further information and should be used in future research.

Fourth, because of the complex structure of multisource designs and our limited sample size, we did not add any moderators or mediators to our models. The list of possible moderators and mediators is long and should be investigated in future research to better understand the complex interaction of leadership-employee processes from multisource perspectives.

Fifth, although the supervisor ratings of their HoL ranged from 1 to 5, mean scores where considerably high and standard deviations were considerably low on all dimensions (*M* = 3.92, *SD* = 0.54 for awareness; *M* = 4.54, *SD* = 0.60 for value and *M* = 3.52, *SD* = 0.75 for behavior). This might have led to variance restrictions on the supervisor level, reducing the possibility to detect interaction effects.

Sixth, our sample consisted of 713 employees nested within 99 teams (supervisors) across 11 different companies from different branches in Southern Germany. On the one hand, this reflects the natural variability of work and work factors and increases external validity. On the other hand, the sample might be too small and heterogeneous to detect significant cross-level interactions (Mathieu et al., 2012).

Finally, we assessed general perceived mental distress as an outcome variable. Prior studies have shown that significant results of multisource relationships appeared only for proximal constructs, which are closely connected to each other (e.g., supervisor-employee relationship and feeling respected and supported), but not for distal constructs, which are further apart from each other [e.g., supervisor-employee relationship and perceived stress; Chen and Tjosvold (2013)]. Thus, general perceived mental distress could be too distal as an outcome and be influenced by many other factors despite leadership quality. Future studies should, therefore, include proximal and distal outcomes to better assess the magnitude of the relationship between multisource assessments of leadership and health outcomes.

### Implications for Practice

Our study showed that HoL ratings significantly differed between supervisors and their staff. A significant relationship between supervisors’ self-perception and the perception of their teams was only found for the behavioral dimension, while this relationship was not significant for the awareness and value dimensions. These findings have important implications for practice. This work could contribute to sensitize employees, supervisors and organizations as a whole that subjective appraisals of HoL might differ from each other. Especially supervisors should be aware that employees’ subjective perceptions of healthy leadership might differ significantly from their own self-perception. The finding, that employees’ subjective perception is highly related to mental health, highlights the importance for supervisors to discuss health topics openly and explicitly in their team and try to create a common understanding of how leadership can contribute to wellbeing at the workplace. For doing so leadership training might be a productive occupational health intervention. However, to date the empirical evidence of the effectiveness of HoL interventions is limited. A recent systematic review found only seven trials of HoL interventions, all of which had only moderate validity and only four of which showed an improvement in health outcomes (Stuber et al., 2020). In addition, our results suggest that training programs should be designed with both leaders and teams in mind. Team interventions for supervisors and their staff could be offered to create a common concept of HoL and mental health in the workplace, thereby reducing discrepancies between the different perspectives (e.g., Ward et al., 2018). This, in turn, should decrease subjectivity biases and might increase self-other agreement and the impact of HoL on employee mental health. Overall, the development of effective HoL interventions represents a central task for future research in the field of occupational health (Rudolph et al., 2020).

## Conclusion

In summary, our results show that for the mental health of employees, their own subjective perception of HoL is relevant, but the supervisors’ self-perception is not. Furthermore, the supervisors’ and employees’ ratings of HoL were significantly related only on the health behavior dimension, but not on the health awareness and value of health dimensions. This shows, that the extent to which employee and supervisor perspectives of HoL correspond to each other is low and may not be well understood, due to its complex nature. Future studies should aim to shed light on the complex processes involved, applying multisource research methods and including theoretically derived moderator variables.

## Data Availability Statement

The raw data supporting the conclusions of this article will be made available by the authors, without undue reservation.

## Ethics Statement

The study was approved by the ethical review committee at the University of Heidelberg, 2017562NMA. The participants provided informed consent to participate in this study.

## Author Contributions

RV and MBi collected the data. RV and NK analyzed the data. RV wrote the first draft of the manuscript. MBo supervised the writing of the first draft. LL supervised the data collection. All authors contributed to conception and design of the study and manuscript revision, read, and approved the submitted version.

## Conflict of Interest

LL received fees for treatment development and providing training. GM is an employee of the sponsor. The remaining authors declare that the research was conducted in the absence of any commercial or financial relationships that could be construed as a potential conflict of interest.
